# Imaging Considerations before and after Liver-Directed Locoregional Treatments for Metastatic Colorectal Cancer

**DOI:** 10.3390/diagnostics14070772

**Published:** 2024-04-05

**Authors:** David-Dimitris Chlorogiannis, Amgad M. Moussa, Ken Zhao, Erica S. Alexander, Constantinos T. Sofocleous, Vlasios S. Sotirchos

**Affiliations:** 1 Department of Radiology, Brigham and Women’s Hospital, Harvard Medical School, Boston, MA 02215, USA; dchlorogiannis@bwh.harvard.edu; 2 Interventional Radiology Service, Memorial Sloan Kettering Cancer Center, New York, NY 10065, USA

**Keywords:** colorectal cancer, liver metastases, margin, thermal ablation, interventional oncology, imaging, locoregional therapy, Yttrium-90 radioembolization, stereotactic body radiation therapy

## Abstract

Colorectal cancer is a leading cause of cancer-related death. Liver metastases will develop in over one-third of patients with colorectal cancer and are a major cause of morbidity and mortality. Even though surgical resection has been considered the mainstay of treatment, only approximately 20% of the patients are surgical candidates. Liver-directed locoregional therapies such as thermal ablation, Yttrium-90 transarterial radioembolization, and stereotactic body radiation therapy are pivotal in managing colorectal liver metastatic disease. Comprehensive pre- and post-intervention imaging, encompassing both anatomic and metabolic assessments, is invaluable for precise treatment planning, staging, treatment response assessment, and the prompt identification of local or distant tumor progression. This review outlines the value of imaging for colorectal liver metastatic disease and offers insights into imaging follow-up after locoregional liver-directed therapy.

## 1. Introduction

Colorectal cancer (CRC) is one of the most frequently diagnosed cancers and the second-most common cause of cancer-related death for men and women combined in the United States [1]. In the past decades, the overall incidence and mortality rates of CRC have declined, which is mainly attributed to the refinement and advancement of screening and available treatment options [1]. However, contemporary evidence points towards a concerning trend in the incidence of CRC in younger patients (<50 years), with current estimations projecting a roughly two-fold increase for patients in their second and third decade of life [2]. Over one-third of the patients with CRC will develop liver metastases, a major contributor to morbidity and mortality [3,4]. Progressing liver metastases can lead to liver dysfunction and failure, either through liver parenchyma replacement, vascular inflow restriction, or biliary obstruction [4].

Hepatic resection has historically been the treatment of choice for resectable colorectal liver metastases (CLMs), though only approximately 20% of patients with CLM are surgical candidates due to significant comorbidities, advanced disease, and/or limited liver functional reserve [5,6]. For this reason, locoregional treatment options including thermal ablation (TA), arterially directed therapies such as Yttrium-90 (^90^Y) transarterial radioembolization (TARE), and stereotactic body radiation therapy (SBRT) have been added to the treatment armamentarium. According to the National Comprehensive Cancer Network Guidelines (NCCN), TA is currently recommended either alone or in combination with surgery when all visible tumors can be treated with clear margins [7,8]. Patients with liver-dominant metastases who are not candidates for resection or ablation can be treated with intra-arterial therapies, including TARE [8,9]. Similarly, SBRT can also be considered for disease not amenable to either surgery or ablation with clear margins [8]. 

Imaging plays a pivotal role in the management of patients with CRC and CLM, from the initial workup and staging to selecting the optimal treatment option, including the suitability for surgical management or thermal ablation in relation to other locoregional treatments. In addition, the evolution in imaging may also offer effective locoregional treatment planning, real-time thermal ablation margin assessment with navigational systems along with 3D confirmation software, as well as positron emission tomography (PET) using 18F-fluorodeoxyglucose (^18^F-FDG) as a radiotracer for the assessment of treatment response and local tumor progression after locoregional therapies [10,11,12,13].

This comprehensive review aims to encapsulate the current clinical evidence regarding the baseline imaging required prior to consideration of liver-directed locoregional treatments, as well as the expected evolution of imaging findings in the treatment zone after the locoregional treatment of patients with CLM. 

## 2. Baseline Imaging Prior to Locoregional Treatment

During the initial workup and staging of CRC, different imaging modalities (CT with and without contrast enhancement [CE-CT], magnetic resonance imaging [MRI], and FDG PET/CT) can be selected for the evaluation of local tumor spread; invasion to adjacent structures; and the involvement of distant sites such as the lungs, liver, and other organs. Within the preceding month prior to any locoregional treatment, updated anatomic and metabolic imaging is recommended to accurately restage the disease [8]. It should be noted that many patients who are being evaluated for potential liver-directed locoregional treatment often have received multiple prior treatments including surgery, hepatic arterial infusion chemotherapy (HAIC), TA, or SBRT, which may make the interpretation of the imaging findings challenging. Moreover, systemic chemotherapeutic agents can also limit the sensitivity of cross-sectional imaging through alterations in the components of the liver parenchyma, like hepatic steatosis, or by lowering the tumor metabolic activity [14,15]. 

A triphasic (unenhanced, arterial, and portal venous phase) CT scan of the chest, abdomen, and pelvis provides the standard option for patient evaluation due to its accessibility and image acquisition speed. CE-CT offers valuable information about tumor anatomy, location, the potential need for contrast administration for tumor localization (during TA), and any other specific guidance or navigation [16,17]. Additionally, it allows extra calculations to be made for efficient treatment planning such as effective liver volume calculation, percutaneous ablation needle trajectory planning, and 3D imaging reconstructions [11,18,19]. CLMs are usually hypodense lesions on unenhanced CT, though they may be isodense to the surrounding liver parenchyma and require contrast administration to increase conspicuity. Following contrast administration, CLMs typically manifest as hypoattenuating lesions with peripheral rim-enhancement (“target sign”) [20]. As such, CLM can also be better seen on the portal venous phase due to the attenuation difference with the surrounding enhancing liver parenchyma. During the arterial phase, it is also possible to identify hypervascular tumors, which may be suitable for combination treatment strategies involving intra-arterial therapies [21]. The reported per-lesion sensitivity of CE-CT is approximately 84%, but for lesions smaller than 1 cm, it significantly decreases to approximately 35% [22]. Lastly, in cases where iodinated contrast is contraindicated due to severe allergy or decreased renal function, alternative imaging modalities such as MRI or FDG PET/CT can be considered [8].

MRI has been gaining a lot of traction in assessing patients with CLM, due to superiority in accuracy, especially for lesions smaller than 1 cm [22]. The diagnostic capacity of MRI can be further increased when used in combination with diffusion-weighted imaging (DWI), gadolinium-based contrast agents, and magnetic resonance cholangiopancreatography (MRCP), with the latter allowing additional evaluation of the relationship and proximity of the target lesions to the central bile ducts [23]. CLMs are usually T1 hypointense and T2 hyperintense tumors with diffusion restriction [24]. Similarly to CT, following contrast administration, CLMs typically demonstrate early peripheral rim-enhancement and a decreased signal in the portal venous and delayed/hepatobiliary phases compared to the surrounding liver parenchyma on T1-weighted MRI, especially when hepatobiliary gadolinium-based contrast agents, such as gadoxetate disodium or gadobenate dimeglumine, are administered. About 50% of the dose of gadoxetate disodium (Eovist^®^, Bayer HealthCare, Whippany, NJ, USA) is excreted through the hepatobiliary pathway and enables the acquisition of functional post-contrast MRCP images, providing a more detailed assessment of the biliary tree [24,25]. The increased diagnostic accuracy of MRI over CT for CLM has been demonstrated through multiple meta-analyses with the per-lesion specificity ranging from 86.9 to 100%, and according to the latest (1.2024) NCCN guidelines for Colon Cancer, liver CE-MRI is recommended over CT to evaluate the exact number and distribution of metastatic foci for local treatment planning, when surgery or liver-directed therapy is considered [8,22,26,27,28]. Definitive evidence on the additional value of CE-MRI and DWI-MRI to CT in the routine investigation of patients with CLM, who are scheduled to undergo local treatment based on CT findings alone, came from the CAMINO study [29]. This international, multicenter, prospective, diagnostic accuracy trial recruited 298 patients with CLM already demonstrated on CE-CT and evaluated the change in the local treatment plan after liver CE-MRI. Notably, 92 (31%) patients exhibited a change in their treatment plan with 40 (13%) changing to more extensive local therapy, 11 (4%) to less extensive local therapy, and 34 (11%) having their curative-intent local therapy withdrawn. In this subgroup, 26 (9%) had disease deemed too extensive and 8 (3%) were found to have benign lesions on liver contrast-enhanced MRI. Lastly, in an economic evaluation study that compared MRI, FDG PET/CT, and CE-CT for the selection of CLM eligible for ablation, the authors found that MRI was the most cost-effective modality [30].

**Figure 1:**
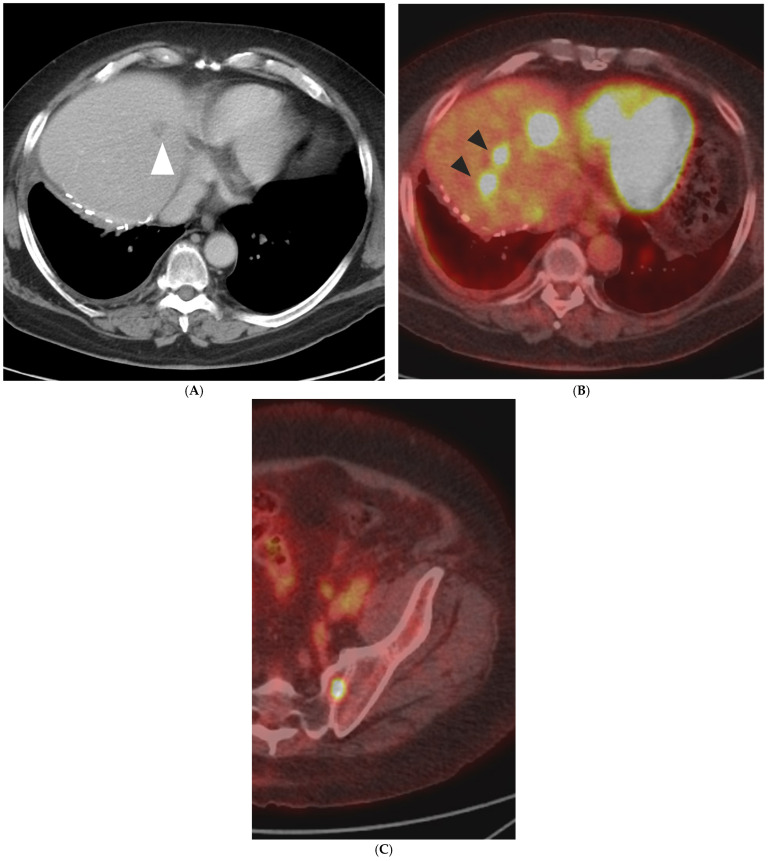
A 75-year-old man with metastatic rectal cancer, prior hepatic arterial infusion pump chemotherapy, and right hepatectomy, referred to interventional radiology clinic for consideration of percutaneous ablation to new 1.7 cm hepatic dome metastasis on CT ((**A**); white arrowhead). Prior to evaluation in clinic, an FDG PET/CT was obtained showing additional sites of intrahepatic disease ((**B**); black arrowheads), as well as osseous metastases ((**C**); left sacrum). The plan for ablation was aborted and systemic chemotherapy was administered.

**Figure 2:**
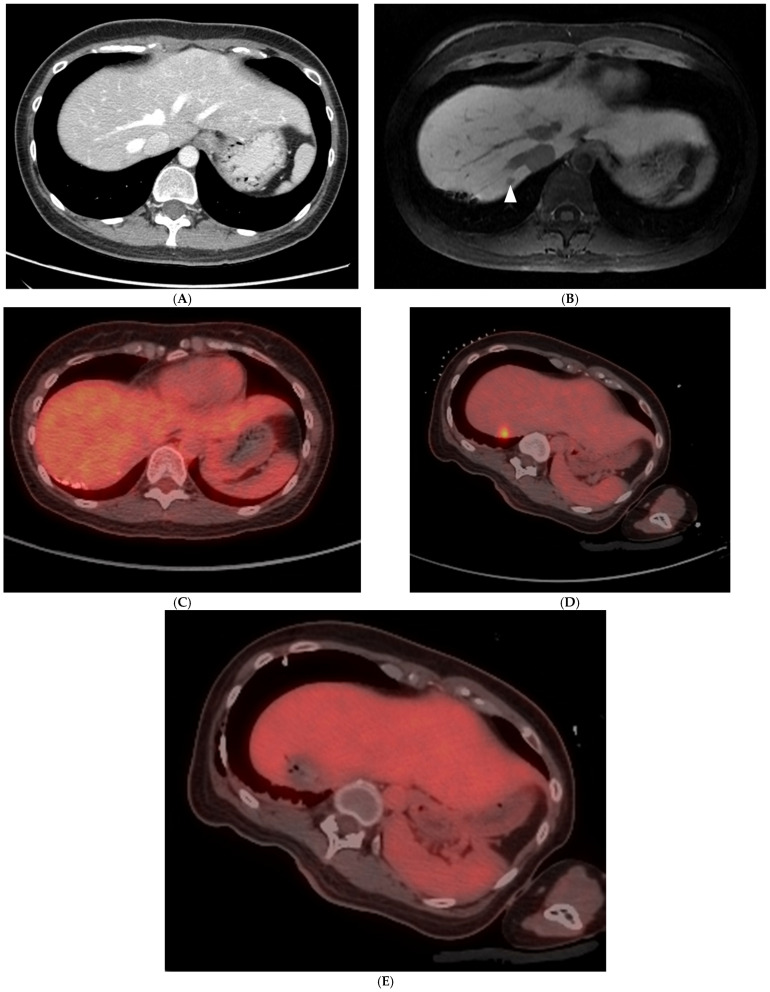
A 39-year-old woman with metastatic rectal cancer, post-liver resection, and hepatic arterial infusion pump placement, on FOLFIRI chemotherapy with solitary 0.7 cm segment 7 metastasis. The metastasis was subtle on the portal venous phase CT (**A**) and conspicuous on the hepatobiliary phase of the MRI ((**B**); white arrowhead). The tumor did not demonstrate increased focal FDG uptake on baseline PET/CT (**C**). Following a 4-week chemotherapy break, the patient presented to interventional radiology for percutaneous microwave ablation. The target demonstrated FDG-avidity after discontinuation of chemotherapy (**D**), permitting PET-guided microwave ablation using the split-dose technique. Immediate post-ablation PET/CT confirms a photopenic defect at the tumor site (**E**).

FDG PET/CT can offer valuable information on the anatomical distribution of CLM, as well as functional insights of their metabolic activity since 18-F-fluorodeoxyglucose is a glucose analog and its uptake correlates with tumor metabolic activity. A semi-quantitative assessment of 18F-FDG activity is commonly conducted using the maximum standardized uptake value of the radiotracer in the tumor (SUVmax). The major advantage of FDG PET/CT lies in its capability to identify distant metastatic lesions as well as difficult-to-detect liver lesions, with data stemming from a meta-analysis reporting a specificity range for CLM of 62% to 74.1% [22,31]. The detection of previously unknown distant or intrahepatic metastases may directly alter the treatment plan (Figure 1). In a meta-analysis of 12 studies by Maffione et al., the pooled change in management of patients with CLM as a result of PET was 24% [32]. Current recommendations do not include routine initial evaluation with FDG PET/CT; however, it may be considered for patients who cannot undergo conventional anatomical imaging and for candidates who may undergo potentially curable surgery or liver-directed therapies [8]. The recommendations also note that FDG PET/CT can be considered for the assessment of treatment response and intrahepatic recurrence after image-guided liver-directed therapies like TA and TARE; therefore, baseline FDG PET/CT prior to these interventions is extremely important and the standard of care at our institution. Some limitations of initial FDG PET/CT imaging include a difficulty in detection of small-size (<10 mm) CLMs or certain mucinous adenocarcinomas, an area where anatomical imaging like MRI is superior [33,34]. Lastly, FDG PET/CT sensitivity has been reported to decrease when evaluating patients who are concurrently receiving chemotherapy [35]. A strategy often employed at the authors’ institution for PET/CT-guided TA of non-FDG avid CLM is to briefly (4–6 weeks) discontinue chemotherapy prior to the intervention, in an effort to increase tumor FDG uptake on the day of the procedure and allow accurate lesion targeting (Figure 2).

## 3. Post-Treatment Imaging and Response Criteria

Evaluating treatment response using a standardized approach is of utmost importance in guiding subsequent therapeutic decisions and predicting the prognosis for patients with CLM after locoregional therapy. The early identification of new or recurrent lesions enables the timely initiation of additional treatments. For this reason, multiple response criteria have been proposed. The “Response Evaluation Criteria In Solid Tumor” (RECIST) criteria use morphologic parameters, such as changes in tumor dimensions, to assess for treatment response [36]. However, these criteria have limitations and often underestimate treatment response after various molecular targeted therapies, such as immune checkpoint inhibitors and bevacizumab regimens [37]. In response to this, many alterations to the RECIST criteria have been proposed, such as the Choi criteria which also encompass changes in tumor attenuation and thus make it more applicable to CLM, due to their hypovascular imaging features on CT [38]. Both, however, do not take into account the metabolic alterations after treatment. Indeed, these changes, as detected on FDG PET imaging, have been directly correlated with tumor response after chemotherapy or locoregional treatments [39,40,41]. Of note, a recent meta-analysis has linked metabolism response after TA and radioembolization treatment with local tumor progression, recurrence-free survival, and overall survival [42]. Similar to RECIST 1.1 and the Choi criteria, two additional sets of criteria are also utilized for quantifying metabolic changes in anti-cancer treatment using FDG PET: the European Organization for Research and Treatment of Cancer (EORTC) criteria and the Positron Emission Tomography Response Criteria in Solid Tumors (PERCIST) [43,44] (Table 1 and Table 2).

## 4. Image-Guided Percutaneous Ablation

Image-guided percutaneous ablation is a widely adopted locoregional treatment option with curative intent using thermal or non-thermal damage. Thermal ablation aims at targeted tumor cell destruction by subjecting them to thermal damage from either high (above 60 °C) or freezing (below −40 °C) temperatures. In the contemporary clinical practice, this can be achieved through a plethora of different thermal modalities, including radiofrequency (RFA), microwave (MWA), cryoablation (CA), and focused ultrasound ablation. Conversely, irreversible electroporation (IRE) is a non-thermal ablation technique utilizing electrical pulses to induce tumor cell death via irreversible membrane cell disruption, while preserving key surrounding structures such as blood vessels and bile ducts [17,45]. 

RFA is the most extensively studied CLM ablation technology, utilizing a high-frequency alternating current (365–500 kHz). The monopolar or bipolar RFA systems induce frictional heating with irreversible protein coagulation occurring between 60 and 100 °C [46,47]. Temperatures exceeding 100 °C cause water evaporation, resulting in tissue desiccation and charring, which limits energy transmission to the surrounding tissues. The limitations of RFA include its relatively small active heating zone (range of approximately a few millimeters), as well as the “heat-sink” effect which limits its efficacy and char residue [46]. MWA operates similarly to RFA by generating heat through electromagnetic currents which eventually also lead to protein denaturation and coagulative necrosis [46]. The main advantages of MWA over RFA include the capability to generate higher and faster intra-tumoral temperatures, leading to the achievement of larger ablation zones within a shorter timeframe, while it is less affected by the heat sink effects and appears to be associated with lower intraprocedural pain [48,49,50]. Cryoablation utilizes extreme cold, typically around −40 °C, to induce cellular damage and death. Cryoablation involves the insertion of one or more cryoprobes into the target lesion with alternating cycles of freezing and thawing [51]. Despite being one of the earliest methods for liver metastases treatment, its use in the liver has been limited [52]. However, a meta-analysis showed comparable safety and efficacy between the three techniques, with MWA yielding more favorable oncologic outcomes compared to RFA and CA [53]. Newer non-thermal ablation technologies, such as histotripsy and pulsed electric fields, are expected to alter the landscape of locoregional treatments in the coming years [54,55,56].

Multiple observational studies and large case series have highlighted several independent factors influencing the technical success of thermal ablation procedures, with adequate ablation margins consistently emerging as the most critical technical factor for local tumor control (often referred to as “A0 ablation”, similarly to the surgical resection devoid of microscopic disease; R0) [7,57,58,59,60]. In a position panel of expert recommendations, an ablation margin of 10 mm has been defined as the ultimate treatment goal when thermal ablation is offered as a potential local cure for CLM [61]. Definitive evidence on the superiority of A0 margins came from a recent meta-analysis which included the pooled results of 21 studies examining the oncological outcomes of thermal ablation stratified by ablation margins. In this study, the authors confirmed that the risk for local tumor progression is at least 3.6 times higher when the minimal ablation margin is less than 5 mm (Risk Ratio: 3.60; 95% CI: 2.58–5.03; *p*-value < 0.001) [62]. When possible, the achievement of an A0 ablation margin is associated with a local tumor progression-free survival exceeding 95%; this represents an opportunity for local cure similar to surgery, but with a reduced morbidity and risk of complications [7]. Thus, assessment of the ablation margin is of paramount importance and must be performed routinely at any institution offering TA for CLM. 

**Figure 3:**
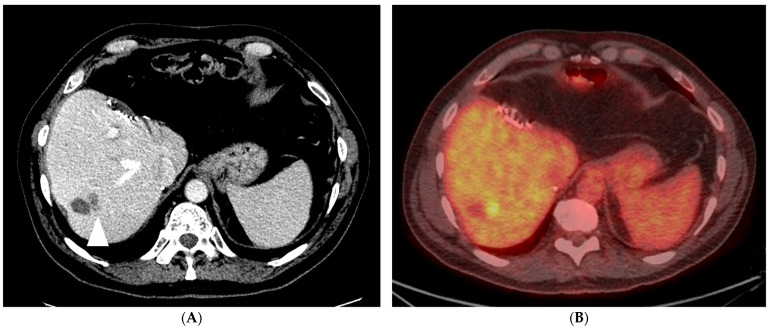
A 67-year-old man with metastatic rectosigmoid cancer, with imaging consistent with local tumor progression, one year after microwave ablation of segment 7 metastasis. Portal venous phase CT shows 1.3 cm hypoattenuating (relative to liver parenchyma) nodule along the medial aspect of the ablation zone ((**A**); arrowhead), with corresponding increased focal FDG-uptake on PET/CT (**B**). The recurrence was treated with repeat microwave ablation.

Historically, the evaluation of the ablation zone and technical efficacy of the ablation session was performed with cross-sectional imaging (CE-CT or CE-MRI) at 4 to 8 weeks, as per the 2014 “Image-guided standardization and reporting criteria” [61]. Within this timeframe, post-ablation imaging confounders such as reactive hyperemia and inflammation would have resolved, allowing the residual unablated tumor to be more conspicuous compared to intraprocedural imaging. During the first month, after percutaneous thermal ablation, ablation zones contain blood products and areas of coagulation necrosis leading to a low-density signal on CT and high T1 as well as low T2 signal on MRI [63]. Transient periablational hyperemia manifests as a thin peripheral rim of enhancement, with an expected resolution within one month [64]. Of note, it is crucial to distinguish this phenomenon from a residual unablated tumor (on first post-ablation follow-up) or local tumor progression (on subsequent follow-up), which typically presents as an irregular peripheral or nodular enhancement within 1 cm of the ablation zone (Figure 3) [64,65]. Moreover, since a technically successful ablation zone is larger than the target tumor, the RECIST criteria cannot be used to assess for ablation efficacy [66]. As such, the first post-ablation imaging serves the new baseline for further assessment of the ablation zone and prompt detection of local tumor progression.

In the current era, there have been remarkable advancements in the field of image fusion software that accurately register cross-sectional imaging scans at different timepoints (before, during, and 4–8 weeks post ablation). Three-dimensional-software assessments of the minimal ablation zone margins are superior to previously described 2D-techniques, in terms of predictive value for local tumor progression [10,65]. Most of these systems rely on either intensity-based deformable image registration or rigid image registration, both of which are prone to inaccurate registration due to liver deformation arising from patient positioning, breathing, protective hydrodissection maneuvers, and local tissue contraction. This phenomenon is attributed to collagen and other protein remodeling after thermal ablation, in combination with profound water evaporation, and tissue dehydration in the ablation zone [67]. Consequently, inspection of the post-ablation imaging underestimates the ablation zone and margins. A comparison of the two registration methods was performed in a retrospective study by Lin et al. in which deformable image registration for minimal ablation margin assessment was found to be more accurate than intensity-based rigid image registration for oncologic outcome prediction in patients who underwent TA for CLM (Area Under the Curve [AUC] 0.9 vs. 0.72, *p*-value < 0.001) [68]. With adequate registration, 3D assessments may also provide intraoperative feedback concerning areas with inadequate coverage of the tumor by the ablation zone, facilitating the need for an overlapping ablation in the same session [10]. The timing of the minimal margin assessment using 3D software has been an ongoing subject of investigation. In a retrospective study by Lin et al., the prognostic accuracy of intraprocedural vs. conventional follow-up timing for 3D minimal ablation margin assessment using CT to predict oncologic outcomes was evaluated [69]. By analyzing the data from 68 patients and 133 ablated CLM, the authors reported a higher AUC for intraprocedural minimal margin quantification vs. initial follow-up CT in predicting 1-year local tumor progression (AUC: 0.89, 95% CI: 0.83–0.94 vs. 0.66, 95% CI: 0.54–0.76, *p*-value < 0.01). Similar results were reported in a study by Zirakchian Zadeh et al. which included 75 patients with 100 CLMs. The intraprocedural 3D assessment of volumes of insufficient coverage by a 5 mm margin exhibited a higher AUC compared to the 4–8-week post-ablation CE-CT for predicting local tumor progression after a median follow-up of 19.6 months (AUC: 0.78 vs. 0.67) [70]. Furthermore, the addition of intraprocedural FDG PET/CT to CE-CT has also been demonstrated by both retrospective and prospective studies to improve targeting accuracy and enable precise endpoint evaluation after TA [10,71,72,73,74]. A recent study by Zirakchian Zadeh et al. with 190 CLMs from 125 patients showed that only 4 (2.1%) CRLMs remained undetected or had poor avidity on intraprocedural PET/CT, while CT alone could not reliably establish the minimal ablation margins in 61 (32.1%) of the cases, highlighting its potential for effective ablation targeting [72]. Additionally, Cornelis et al. highlighted the predictive value of immediate FDG PET-CT for local tumor progression prediction during the first year after TA in comparison to immediate CE-CT, serving as a possible surrogate imaging biomarker for this cause [13,71]. 

There is no consensus on the imaging modality or the optimal time intervals between subsequent follow-up imaging assessments after TA. After the new baseline cross-sectional imaging, follow-up is usually performed every 2–4 months, at least for the first year after ablation. In this context, a meta-analysis by Samim et al. compared the diagnostic performance of FDG PET/CT, CT, and MRI for the timely detection of disease progression following TA [75]. By pooling the results from 10 studies, the investigators found that the pooled sensitivity of FDG PET/CT was significantly higher than that of CT (84.6%, 95% CI: 75.0–90.6 vs. 53.4% 95% CI: 29.0–76.4, *p*-value < 0.05), while no difference was found in the pooled specificity between the two (92.4%, 95% CI:86.5–95.9 vs. 95.7%, 95% CI: 87.5–98.6, *p*-value > 0.05). With only two studies reporting results about the diagnostic accuracy of MRI, a direct comparison with the other two modalities could not be performed. However, in a prospective diagnostic accuracy evaluation for local tumor progression after RFA for CLM, FDG PET/CT and MR imaging were comparable in terms of accuracy (86 vs. 91%, *p*-value >0.05) [76]. Similar results about the utility of FDG PET were also demonstrated in a meta-analysis by Bijstra et al. which highlighted its indispensable value for oncologic outcome monitoring [42]. Thus, current NCCN guidelines recommend FDG PET/CT for monitoring the response and intrahepatic recurrence after image-guided liver-directed therapies [8]. At the authors’ institution, the standard imaging follow-up protocol after TA includes at least one anatomic cross-sectional modality (triphasic CE-CT and/or MRI, preferably with Eovist^®^), as well as FDG PET/CT, initially at 4–8 weeks to assess TA efficacy and every 2–4 months thereafter, for at least the first year.

## 5. Yttrium-90 Transarterial Radioembolization

Transarterial radioembolization (TARE) with Yttrium-90 (^90^Y) is an arterially directed locoregional therapy that involves the administration of resin or glass microspheres [77]. The microspheres loaded with the beta-emitting ^90^Y radioisotope are selectively delivered into the hepatic artery branches, optimizing radiation exposure to the tumor and minimizing injury to the surrounding liver parenchyma, which is predominantly supplied by the portal vein [78]. TARE is typically offered to patients with liver-dominant colorectal cancer metastases who are not candidates for surgery or ablation. The oncologic outcomes after the addition of TARE to systemic chemotherapy have been the subject of extensive research. A combined analysis aimed to evaluate the results of multiple large randomized controlled trials in which TARE was added to the first-line chemotherapy FOLFOX regimen [79]. The authors highlighted that even though a significant improvement in liver disease control was noted in the combination treatment arm, this did not translate into an overall survival benefit. Similarly, the oncologic outcomes of the addition of TARE to second-line chemotherapy alone were recently examined in a phase III randomized controlled trial that included 428 patients with CLM who failed first-line systemic treatment (EPOCH trial) [80]. The investigators demonstrated statistically significant, higher objective response rates (34% vs. 21.1%) and longer progression-free and liver progression-free survivals (median 8.0 and 9.1 months vs. 7.2 and 7.2 months, respectively) in the combination arm (TARE + chemotherapy), compared to chemotherapy alone. Though overall survival was not a primary endpoint of this study, there was no difference between the two arms (14.0 vs. 14.4 months, Hazard Ratio: 1.07, 95% CI, 0.86–1.32). As such, current NCCN guidelines for colorectal cancer recommend TARE in carefully selected patients with liver-dominant chemorefractory metastases [8].

Immediate (within hours) post-treatment imaging is obtained to confirm the treatment zone and ensure that non-target radiation delivery did not occur. Bremsstrahlung Single-Photon Emission Computed Tomography (SPECT)/CT is commonly used for this assessment; however, it suffers from limited image quality and spatial resolution. ^90^Y also emits a small number of positrons (0.003%), permitting visualization with PET [79]. PET has been shown to be superior to Bremsstrahlung SPECT due to its higher spatial resolution [81]. Both modalities can be used for dosimetry analysis and calculation of the absorbed tumor dose and tumor-to-normal-liver ratio [79,80,81]. The response after TARE is characterized by several findings, including a reduction in tumor size, tumor necrosis, and devascularization/hypoenhancement [82]. An increase in tumor size at early follow-up should be interpreted with caution, as treatment-induced intra-tumoral edema and hemorrhage can manifest as pseudoprogression. Therefore, CT or MRI may take up to 2 to 3 months to accurately assess the treatment response [82,83]. A peripheral rim-enhancement pattern similar to post-ablation hyperemia may represent post-treatment fibrosis rather than recurrence. The key difference is that with a viable tumor, the enhancing component increases on serial scans and may warrant the administration of additional treatments [84]. DWI-MRI can also aid in distinguishing reactive edema from residual tumor or local tumor progression [85]. TARE can also affect the normal liver parenchyma by inducing ischemia and hepatitis, producing an irregular patchy enhancement in the treated liver volume, thereby complicating the accuracy of treatment response [84]. Finally, post-procedural complications such as hepatic fibrosis leading to portal hypertension may be observed during follow-up [84]. 

**Figure 4:**
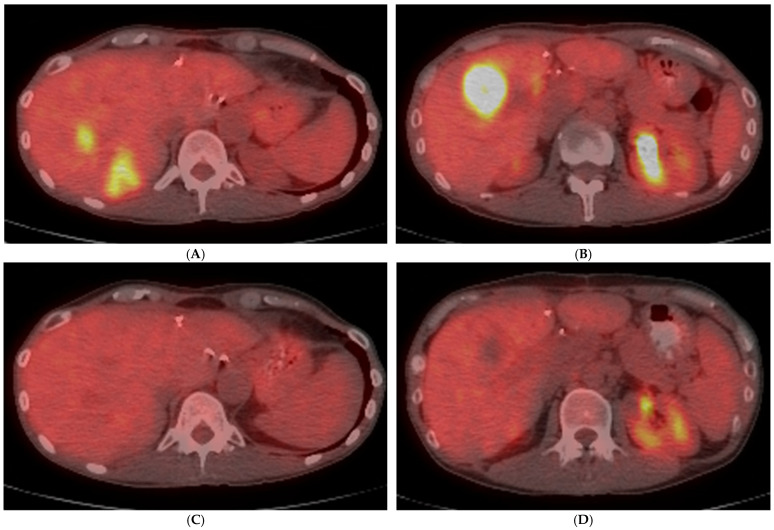
A 52-year-old woman with liver-dominant metastatic colon cancer, with progressing liver metastases despite hepatic arterial infusion chemotherapy and systemic chemotherapy. Baseline PET/CT demonstrated multiple FDG-avid tumors in the right hepatic lobe (**A**,**B**). Following Yttrium-90 radioembolization, follow-up PET/CT after 5 weeks confirmed complete metabolic response (**C**,**D**). Based on size criteria alone (RECIST 1.1), this would have been considered stable disease.

FDG PET/CT is also recommended for early disease monitoring since a metabolic response can be visible within 4–6 weeks after TARE, prior to CT or MRI (Figure 4) [9,86]. This is particularly important for CLM requiring staged bilobar TARE because FDG PET/CT can allow the earlier identification of response in the treated lobe. If disease control (stable metabolic disease, partial metabolic response, or complete metabolic response) has been achieved in the treated liver volume and radiosensitive disease has been documented, proceeding with the second session of TARE to the opposite lobe can be justified. Conversely, if disease progression has occurred in the initial treatment volume, proceeding with the second treatment risks exposing additional liver parenchyma to toxicity with questionable benefit in terms of tumor control [9]. 

After TARE for CLM, multiple response criteria have been assessed. In a retrospective study which included 25 patients and 46 CLMs who underwent TARE, the RECIST 1.1, Choi criteria, tumor attenuation criteria, and EORTC PET criteria were assessed for correlation with treatment response and predictors for intrahepatic progression. The authors reported that RECIST 1.1 had poor sensitivity for detecting metabolic responses by EORTC PET criteria, whereas there was a link between SUVmax and tumor attenuation in CT (measured by Hounsfield units). The EORTC PET criteria, Choi criteria, and tumor attenuation criteria were equally reliable surrogate imaging biomarkers of liver progression-free survival after TARE [87]. Similarly, patients with an FDG PET/CT metabolic response according to PERCIST criteria also have better predicted overall survival [86,87,88]. 

## 6. Stereotactic Body Radiation Therapy (SBRT)

Stereotactic body radiation therapy (SBRT) is a subtype of external beam radiation therapy (EBRT) which delivers high doses of radiation to a tumor, usually over a small number of sessions. It utilizes advanced imaging techniques for effective treatment planning to precisely target tumors, while mitigating the adverse effects of radiation to proximal healthy tissue [89]. In a phase II trial of 76 patients with unresectable CLM, the 5-year local control rate was 75% with the overall survival being 18% [90]. Current NCCN guidelines for colorectal cancer recommend SBRT in selected patients who cannot undergo either surgery or thermal ablation [8]. 

**Figure 5:**
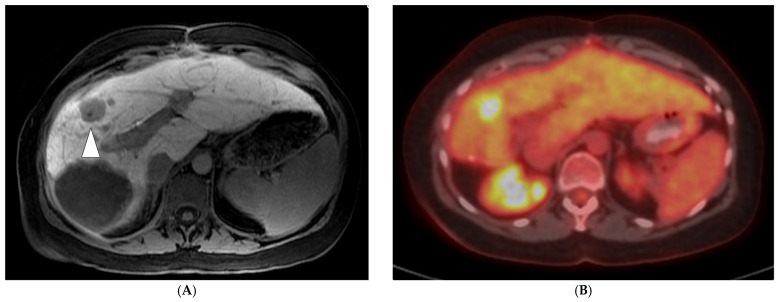

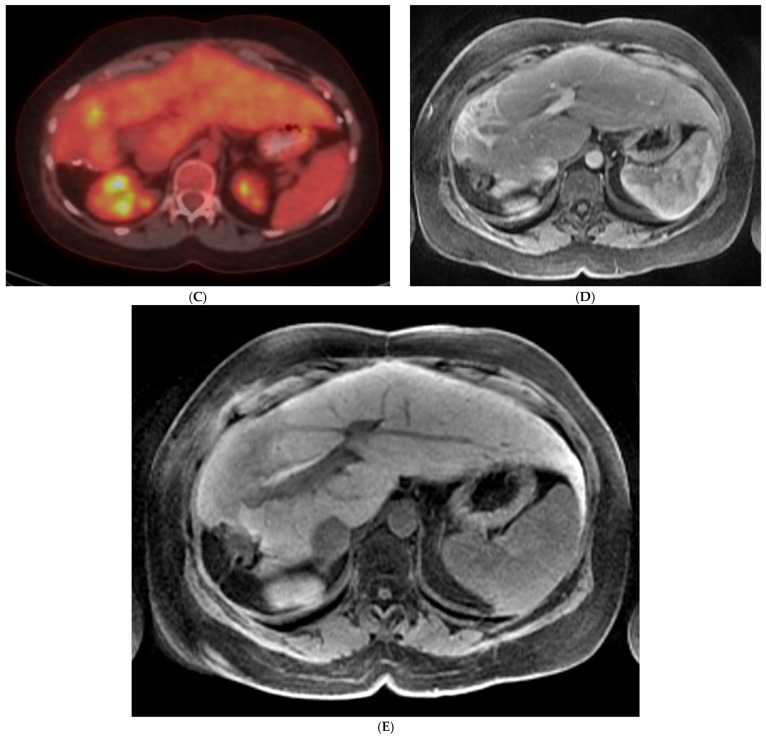
A 47-year-old woman with metastatic rectal cancer, status post chemotherapy, primary tumor, and posterior sector liver resection, with 2.9 cm segment 5 liver metastasis, as seen on the hepatobiliary phase of baseline MRI ((**A**); white arrowhead). This was treated with SBRT (6000 cGy in 5 fractions). PET/CT after 4 months showed persistent uptake in the treatment zone ((**B**); SUVmax 6.1; liver SUVmean 2.8). After 9 months, there was a continued decrease in FDG uptake in the treatment zone, above that of background liver ((**C**); SUVmax 3.4; liver SUVmean 2.4). MRI on the same day showed geographic area of arterial enhancement (**D**), with decreased signal on the hepatobiliary phase (**E**).

Imaging after SBRT to detect differences in tumor size or attenuation patterns is usually performed with multiphasic CE-CT or CE-MRI every 3 months. Earlier imaging may be unreliable as radiation-induced changes take time to develop [91]. Between 3 and 6 months, the peritumoral irradiated liver parenchyma may show arterial hyperenhancement, which corresponds to a focal liver reaction to radiation [92]. The CLM size response after SBRT has a wide variability but is usually characterized by a decrease in enhancement observed on CT or MRI, especially within the first 4 months [93]. However, since the enhancement patterns may persist even beyond the 1-year follow-up, these should be interpreted with caution and differentiated from tumor progression [94]. After 6 months, expected tumoral changes include continuation of the decrease in size and enhancement as well as the reduction in or involution of the focal liver reaction, which is reduced to fibrotic and atrophic changes. Additionally, a peritumoral-rim enhancement pattern may also be seen after SBRT therapy for CLM and can resemble local tumor progression requiring a holistic evaluation with additional laboratory biomarkers to aid in the definitive diagnosis (Figure 5). A restrictive pattern on DWI-MRI can assist in this differentiation, as this is typically observed with a residual or recurrent tumor [95]. 

Metabolic imaging can also be utilized to monitor tumor response after SBRT, though there is a paucity of dedicated data in the literature. A study reviewing changes on PET after SBRT to 35 solid liver metastases reported an estimated SUVmax decay half-time of 2.0 months, with the SUVmax approaching a nadir similar to the background liver after approximately 5 months in controlled tumors [96]. Thus, the metabolic response documented by FDG PET/CT may present on cross-sectional imaging before early anatomical changes [96]. In contrast, local treatment failures were considered tumors with an SUV max > 6, after a prior post-SBRT value below that threshold [96]. Of note, low levels of increased tracer uptake may persist in the treatment zone, as a result of ongoing tissue repair [96].

## 7. Conclusions

Imaging is an integral component in the management of patients with colorectal liver metastases, prior to and after locoregional treatment with thermal ablation, transarterial radioembolization, and stereotactic body radiation therapy. While CT is the cornerstone imaging modality for the initial workup, staging, monitoring, and surveillance, MRI can also provide more details due to its higher diagnostic accuracy, while the role of FDG PET/CT has been expanding and may be necessitated for follow-up imaging, especially after thermal ablation and radioembolization. 

## Figures and Tables

**Table 1:** Characteristics of commonly used treatment response criteria after locoregional therapy for colorectal liver metastasis (Abb: RECIST 1.1: Response Evaluation Criteria in Solid Tumors, EORTC: European Organization for Research and Treatment of Cancer, PERCIST: Positron Emission Tomography Response Criteria in Solid Tumors).

Criteria	Imaging Modality	Key Features	Tumor Response Categories
RECIST 1.1 [36]	CT/MRI	Changes in tumor size	Complete Response, Partial Response, Stable Disease, Progressive Disease
Choi Criteria [38]	CT/MRI	Changes in tumor size and attenuation	Complete Response, Partial Response, Stable Disease, Progressive Disease
EORTC-PET Criteria [43]	PET	Changes in tumor FDG uptake	Complete Metabolic Response, Partial Metabolic Response, Stable Metabolic Disease, Progressive Metabolic Disease
PERCIST [44]	PET	Changes in tumor FDG uptake	Complete Metabolic Response, Partial Metabolic Response, Stable Metabolic Disease, Progressive Metabolic Disease

**Table 2:** Classification of treatment responses based on commonly used criteria after locoregional therapy for colorectal liver metastasis (Abb: RECIST 1.1: Response Evaluation Criteria in Solid Tumors, EORTC: European Organization for Research and Treatment of Cancer, PERCIST: Positron Emission Tomography Response Criteria in Solid Tumors).

Tumor Response	RECIST 1.1 [36]	Choi Criteria [38]	EORTC-PET Criteria [43]	PERCIST [44]
Complete Response	Disappearance of all target lesions	Disappearance of all enhancing target lesions	Complete resolution of ^18^F-FDG uptake in all lesions	Complete resolution of ^18^F-FDG uptake in all lesions
Partial Response	≥30% decrease in sum of longest diameter of target lesion	≥15% decrease in tumor attenuation (HU) or ≥10% decrease in tumor size	Decrease in SUVmax of >25%	≥30% decrease in the SUVmax of target lesions
Stable Disease	Neither sufficient features to qualify as a partial response nor progressive disease	Neither sufficient features to qualify as a partial response nor progressive disease	Neither sufficient features to qualify as a partial response nor progressive disease	Neither sufficient features to qualify as a partial response nor progressive disease
Progressive Disease	≥20% increase in the sum of the longest diameters of target lesions or appearance of new lesions	≥10% increase in tumor size or a decrease in tumor density < 10%, or appearance of new lesions	≥25% increase in SUVmax or appearance of new FDG-avid lesions	≥30% increase in the SULpeak of target lesions or appearance of new FDG-avid lesions

## Data Availability

Not applicable.
